# *Salmonella stanley* ovarian abscess with endometrioma in a pregnant woman in the third trimester

**DOI:** 10.1016/j.idcr.2021.e01068

**Published:** 2021-03-10

**Authors:** Hirokazu Toyoshima, Miki Hagimoto, Motoaki Tanigawa, Hiroyuki Tanaka, Yuki Nakanishi, Shigetoshi Sakabe

**Affiliations:** a Department of Infectious Diseases, Japanese Red Cross Ise Hospital, Ise, Japan; b Department of Obstetrics and Gynecology, Mie University Hospital, Tsu, Japan; c Department of Respiratory Medicine, Japanese Red Cross Ise Hospital, Ise, Japan

**Keywords:** *Salmonella stanley*, Ovarian abscess, Endometrioma, Pregnancy

## Abstract

Ovarian abscesses infected with *Salmonella* species are a rare clinical occurrence. A 26-year-old woman at 36 weeks gestation presented with high fever, left lower abdominal pain, and diarrhea; she was referred to our hospital 5 days after a vaginal delivery. She was diagnosed with a left ovarian abscess and underwent abscess enucleation. According to the stool findings, we speculated that the abscess was infected with *Salmonella* species; appropriate antibiotics were administered after the positive identification of *Salmonella stanley*. This case highlights the importance of treating infections in pelvic abscesses appropriately to limit the effects on fertility.

## Highlights

• *Salmonella stanley* can be identified according to the Kauffman-White scheme.
• Ovarian abscesses without tubal involvement can occur as metastatic infections.
• Th2 cytokine dominance in pregnant women increases susceptibility to *Salmonella*.

## Introduction

A tubo-ovarian abscess is commonly associated with pelvic inflammatory disease (PID) and is one of the major complications of acute PID. It occurs in 10–15 % of hospitalized women with PID [1]. Generally, tubo-ovarian abscess occurs secondary to salpingo-oophoritis. However, isolated ovarian abscesses without tubal involvement can occur as metastatic infections, especially as superinfected ovarian cysts [2]. We report a case of *Salmonella stanley* ovarian abscess without fallopian tube abscesses in a pregnant woman with endometrioma. Additionally, we reviewed previous reports of non-pregnant and pregnant women with *Salmonella* ovarian abscesses with superinfected endometriomas. Furthermore, we evaluated the relationships between *Salmonella* infections, preexisting endometriomas, and pregnancy. This report emphasizes the potential of ovarian cysts and endometriomas to become superinfected with *Salmonella*, especially in pregnant women, and underscores the importance of well-timed clinical and microbiological diagnoses to prevent later infertility.

## Case

**Fig. 1:**
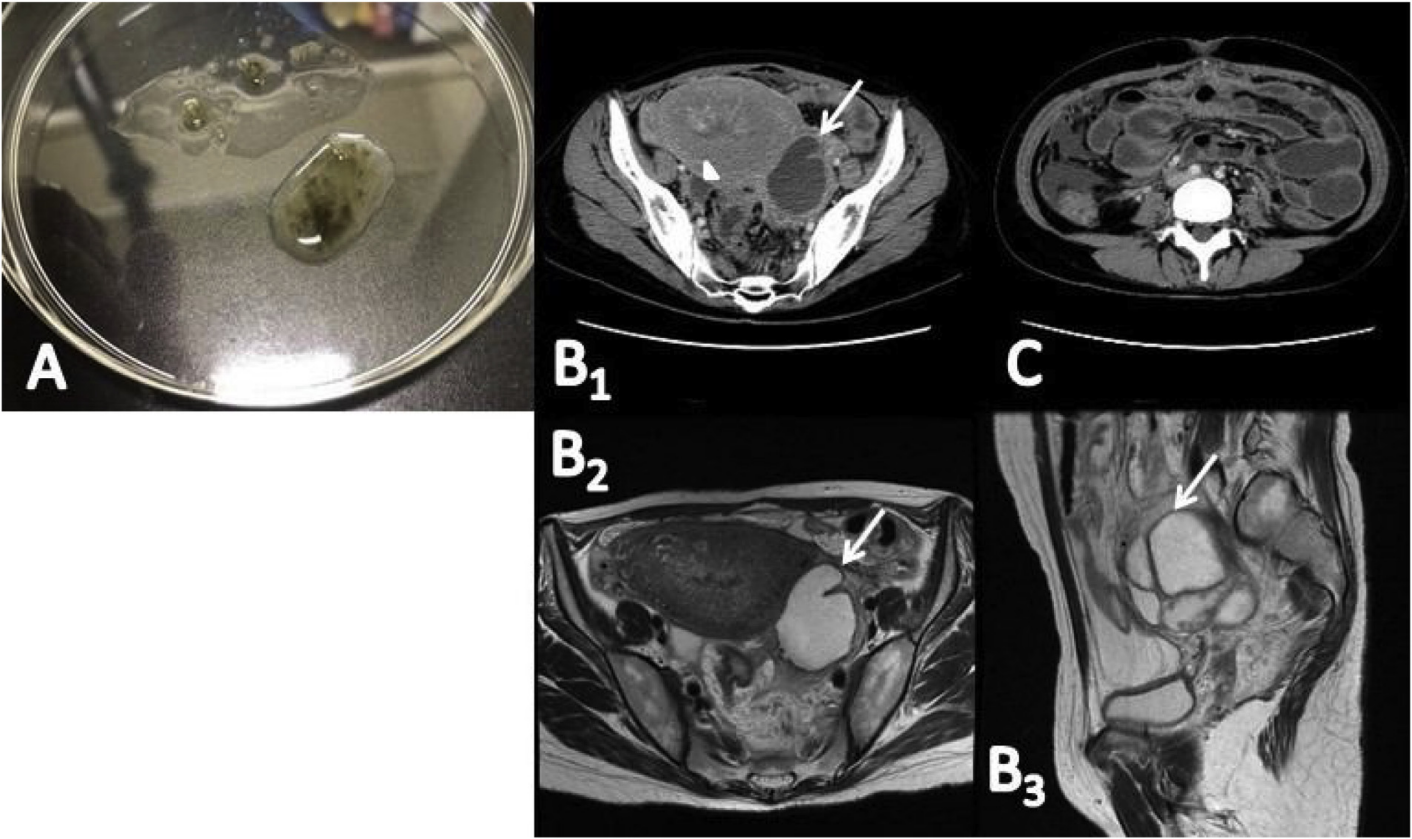
Stool and CT findings. (A): A sample of greenish diarrhea. (B_1-3_): CT and magnetic resonance images showing a left adnexal mass (arrows) and fluid collection (arrow) in the pelvis. (C): CT image also showing wall thickness and fluid collection in the small intestine. CT, computed tomography. (For interpretation of the references to colour in this figure legend, the reader is referred to the web version of this article).

A 26-year-old Japanese woman, gravida 1, para 0, at 36 weeks gestation with an unremarkable prenatal course presented with high fever, shaking chills, left lower abdominal pain, and diarrhea; therefore, she was referred to our hospital 5 days after a vaginal delivery. She had been treated with cefmetazole 1 g every 12 h for 5 days at another hospital. She presented with nausea, greenish diarrhea (Fig. 1A), and one vomiting episode along with the abovementioned symptoms at our hospital. She had no history of traveling abroad or contact with improperly handled foods or animals during pregnancy and fertility treatments. She had no known allergies and had not been prescribed any recent medications.

She was alert, and her vital signs were: body temperature, 39.3 °C; blood pressure, 123/73 mmHg; heart rate, 94 beats/minute; respiratory rate, 20 breaths/minute; and percutaneous oxygen saturation, 98 % on room air. Physical examination showed hypoactive bowel sounds on auscultation and abdominal tenderness mainly in the lower abdomen. Other examinations were unremarkable. Laboratory findings were: total protein, 5.5 g/dL; albumin, 1.8 g/dL; alanine aminotransferase, 5 IU/L; aspartate aminotransferase, 10 IU/L; lactate dehydrogenase, 122 IU/L; blood urea nitrogen, 7 mg/dL; creatinine, 0.51 mg/dL; C-reactive protein, 18.88 mg/dL; white blood cell count, 10,700/μL with 88.8 % neutrophils; hemoglobin, 9.5 g/dL; and platelet count, 30.1 × 10^4^/μL.

**Fig. 2:**
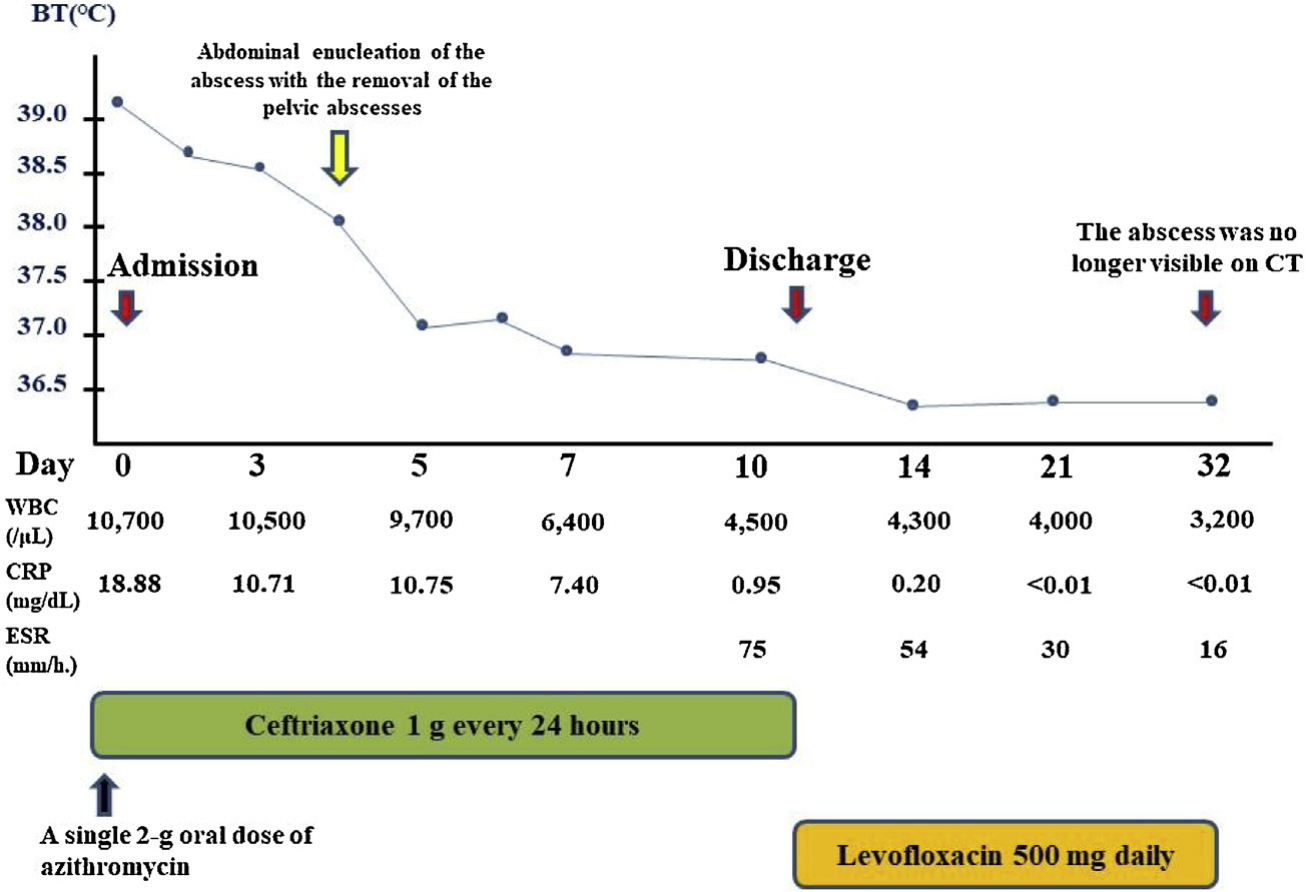
Clinical course in this case. BT, body temperature; CT, computed tomography; WBC, white blood cell count; CRP, C-reactive protein; ESR, erythrocyte sedimentation rate. (For interpretation of the references to colour in this figure legend, the reader is referred to the web version of this article).

Sonographic examination showed a left multicystic adnexal mass measuring 8 × 7.5 cm. Computed tomography (CT) and magnetic resonance imaging (MRI) showed a fluid collection in the pelvis and wall thickness and fluid collection in the small intestine (Fig. 1B, C). She was diagnosed with a left ovarian abscess with pelvic peritonitis and inflammation of the small intestine. Blood and stool cultures were taken, and intravenous ceftriaxone 1 g every 24 h and a single 2-g oral dose of azithromycin were administered. Subsequently, she underwent abdominal enucleation of the abscess with removal of the pelvic abscesses on day 4 post-admission. We found pelvic peritonitis with several abscesses; the bilateral fallopian tubes were intact. Cultures of the abscesses were positive for *Salmonella* species susceptible to ampicillin (minimum inhibitory concentration [MIC] ≤2 μg/mL), ceftriaxone (MIC ≤ 1 μg/mL), ciprofloxacin (MIC ≤ 0.06 μg/mL; susceptibility defined as MIC ≤ 0.06 μg/mL), and levofloxacin (MIC ≤ 0.06 μg/mL; susceptibility defined as MIC ≤ 0.12 μg/mL) according to E-tests (bioMérieux, Marcy I’Etoile, France) (Table 1). The VITEK II system (bioMérieux) indicated the *Salmonella* group, while *S. enterica* subsp. *enterica* was identified by 16S rRNA gene sequencing. Isolate serotyping was performed by latex agglutination using mono- and poly-valent anti-sera for O and H antigens according to the Kauffman-White scheme. The serovar was determined to be *S. stanley* (O4:d:1,2). Blood and stool cultures were negative. Pathology of the left ovary revealed endometrioma with acute inflammation and abscess. She was treated with ceftriaxone for 7 days followed by oral levofloxacin 500 mg daily for 3 weeks postoperatively according to the susceptibility test (Fig. 2). Consequently, the abscess was no longer visible on CT, and the patient has remained well.

**Table 1:** Antimicrobial susceptibility test results.

*Salmonella stanley*		
Antimicrobial agents	MIC (μg/mL)	Interpretation of susceptibility
Ampicillin	≤2	S
Sulbactam/ampicillin	≤2	S
Cefazolin	≤4	R
Cefotiam	≤8	R
Cefmetazole	≤1	R
Cefotaxime	≤1	S
Ceftriaxone	≤1	S
Ceftadizime	≤1	S
Cefepime	≤1	S
Imipenem/cilastatin	≤0.25	S
Meropenem	≤0.25	S
Gentamicin	≤1	R
Amikamycin	≤2	R
Sulfamethoxazole/trimethoprim	≤1/19	S
Nalidixic acid*		S
Ciprofloxacin	≤0.06	S
Levofloxacin	≤0.06	S

*Interpreted according to the disk method. MIC, minimum inhibitory concentration; S, susceptible; R, resistant.

## Discussion

This report highlights the potential of ovarian cysts and endometriomas to become superinfected with *Salmonella* in pregnant women and the importance of timely clinical and microbiological diagnoses to prevent later infertility.

*Salmonella*-infected ovarian abscesses are rare. However, *Salmonella* species can cause extraintestinal infections that may not be suspected in the setting of mild primary infection. Extraintestinal infections include orthopedic infections, endovascular infections, visceral abscesses of any organ, and ovarian endometriomas superinfection (as in our case). Nine English-language reports including pathologically diagnosed superinfected ovarian endometriomas caused by *Salmonella* infection have been published since 1963 [[2], [3], [4], [5], [6], [7], [8], [9], [10]]. These reports, plus this case, are summarized in Table 2. Blood cultures were positive in only 3 (30 %) of 10 patients. Generally, *Salmonella* infections can cause isolated ovarian abscesses without tubal involvement as metastatic infections. Therefore, we cannot exclude *Salmonella* extraintestinal infections based on negative blood cultures. Furthermore, *Salmonella* ovarian abscesses are presumed to result in hematogenous spread to endometriomas or ovarian cysts, including dermoid cysts and teratomas [11]. However, only 2 (20 %) of 10 patients were diagnosed with endometriomas before admission. Thus, diagnostic imaging (e.g., ultrasonography, CT, and MRI) is needed to diagnose superinfected ovarian cysts, including endometriomas. Here, it was probable that the patient had an undiagnosed preexisting ovarian endometrioma that predisposed her to *S. stanley* multiplication via the bloodstream following transient and undetected bacteremia.

**Table 2:** Superinfection of endometriomas.

Authors	Age (years)	Comorbidity	Location	Preceding symptoms	Positive blood cultures	Positive stool cultures	Pathogens	Tubal involvement	Pregnancy	Size (cm)	Antibiotic used	Antibiotic duration (days)	Outcome
Adelman et al. [2]	28	None	USA	Diarrhea, abdominal pain, fever, anorexia	Positive	Positive	*S. schwarzengrund*	None	No	12	Ciprofloxacin Metronidazole Ceftriaxone	14	A
Magliulo et al. [3]	30	None	Italy	Abdominal pain, fever	Negative	Negative	*S. brandenburg*	None	No	11	Chloramphenicol	10	A
Ghose et al. [4]	28	None	UK	Bloody diarrhea, abdominal pain, vaginal bleeding	Negative	Negative (positive after surgery)	*S. stanley*	Yes	No	10 × 8×3	Amoxicillin Cotrixazole	19	A
Kemmann et al. [5]	26	Endometrioma	Canada	None	Negative	Negative	*Salmonella* spp.	None	No	7	Ciprofloxacin	14	A
Li and Cohen [6]	31	SLE	Hong Kong	None	Positive	Positive	*S. enteritidis* (blood, stool)/*S. typhimurium* (pus)	Yes	No	4	Ciprofloxacin Ofloxacin	28	A
Burgmans et al. [7]	16	None	Netherlands	Abdominal pain, fever, headache, fatigue, productive cough	Negative	Negative	*S. enteritidis*	None	No	8.5 × 7.8	Ofloxacin	11	A
Wang et al. [8]	43	None	Taiwan	Abdominal fullness, nausea, vomiting, diarrhea, fever	Positive	NA	*S. enteritidis*	None	No	25 × 20	Ampicillin Ceftriaxone	35	A
Thaneemalai et al. [9]	38	Endometrioma	Kuala Lumpur	Fever, diarrhea, vaginal discharge	Negative	Negative	*S. enteritidis*	Yes	No	8 × 4	Cefuroxime Metronidazole	7	A
Kudesia and Gupta [10]	32	None	USA	Fever, abdominal pain, bloody diarrhea	Negative	Negative	*S. corvalis*	None	No	15	Ceftriaxone	42	A
This case	26	None	Japan	Diarrhea, abdominal pain, fever, nausea, vomiting	Negative	Negative	*S. stanley*	None	Yes	8	Ceftriaxone Azithromycin	32	A

SLE, systemic lupus erythematosus; NA, not applicable; A, alive; UK, United Kingdom; USA, United States of America.

*Salmonella* gastroenteritis can cause greenish diarrhea and usually results from contact with improperly handled food. It can also be acquired via the fecal-oral route between humans and animals. However, few cases of *Salmonella* infections associated with improperly handled foods or close contact with animals have been reported. Only the cases of one patient with a history of consuming a takeaway meal [4] and another with a history of exposure to camels [2] have been published. Hence, we cannot exclude *Salmonella* infections based on a history that does not include improperly handled foods or close contact with animals. Here, we speculated that the patient had *Salmonella* superinfection of the left endometrioma according to the presentation of greenish diarrhea and preoperative CT findings, despite negative blood and stool cultures and no history of contact with improperly handled foods or animals. Additionally, we surmised that the preceding cefmetazole administration at another hospital partially accounted for the negative blood and stool culture results. Although cephamycins, including cefmetazole, may be microbiologically active (MIC ≤ 1 μg/mL here), they are not clinically effective according to the Clinical and Laboratory Standards Institute criteria [12]. Furthermore, stool cultures were positive in only two (22 %) of nine patients (Table 2), indicating that we cannot exclude *Salmonella* extraintestinal infections based on negative stool cultures with or without preceding antibiotic administration. Symptom frequency in the previous reports was as follows: fever (70 %), lower abdominal pain (60 %), diarrhea (60 %), and nausea or vomiting episodes (20 %) (Table 2). These symptoms may indicate adnexal infections; however, they are not specific to ovarian abscesses (e.g., pyelonephritis). Etiologies of ovarian abscesses have been reported as direct contamination by fine-needle aspiration [13], hematogenous bacterial spread from a urinary tract infection [14], and association with a gastrointestinal *Salmonella* infection [3,4]. Our patient’s symptoms started with abdominal pain, greenish diarrhea, and high fever, and CT revealed enteritis. Thus, we speculated that the small intestine was the most plausible infection source.

The salmonellosis rate in pregnant women is the same as that in the general population (i.e., 0.2 %) [15]. However, *Salmonella* species can cause more severe infections in older people, infants, those with human immunodeficiency virus infection, those undergoing treatment with immunosuppressive agents, patients with malignancy, organ transplant recipients, or pregnant women [15]. During pregnancy, the immune system must protect itself without rejecting foreign paternal antigens. The immune system resolves this dilemma by altering the Th1/Th2 cytokine level to Th2 cytokine dominance [16], which protects the fetus from the Th1-mediated immune system at the fetal-maternal interface. Nevertheless, this also makes pregnant women susceptible to *Salmonella* infections because it is an intracellular pathogen and its immunity is Th1 dependent [16].

Three reports of *Salmonella* ovarian abscesses in pregnant women [[17], [18], [19]] plus this case are summarized in Table 3. All patients had ovarian abscesses in the third trimester, and two patients had been diagnosed with dermoid cysts before admission. It is unclear why all the patients had *Salmonella* ovarian abscesses in the third trimester. Generally, immune system modulation during pregnancy contributes to differential responses that depend on both microorganisms and the pregnancy stage. These findings indicate the tendency of *Salmonella* spp. to cause ovarian abscesses in pregnant women in the third trimester (as with *Listeria monocytogenes*) [20]. The symptom frequency in the patients with *Salmonella* ovarian abscess was fever (75 %), lower abdominal pain (75 %), diarrhea (75 %), and nausea or vomiting episodes (50 %). Blood cultures were positive in one (25 %) of four patients. In this review, the percentage of positive blood cultures in pregnant women with *Salmonella* ovarian abscesses was low, similar to that in patients with *Salmonella* ovarian abscesses with preexisting endometriomas (25 % vs. 30 %). Regarding serotypes, the most common serovar was Enteritidis, which was identified in 4 of 10 cases (40 %) of *Salmonella* superinfections of ovarian endometriomas (Table 2). Contrastingly, there have been no reports of *S. stanley* ovarian abscesses in pregnant women (Table 3). This is the first report of an *S. stanley* ovarian abscess without fallopian tube abscesses in a pregnant woman with endometrioma.

**Table 3:** Ovarian abscesses in pregnant women.

Authors	Age (years)	Comorbidity	Location	Preceding symptoms	Positive blood cultures	Positive stool cultures	Pathogens	Tubal involvement	Gestational weeks	Size (cm)	Antibiotic used	Antibiotic duration (days)	Outcome
Brelje and Garcia-Bunuel [17]	26	Dermoid cyst (infected)	USA	Increased urinary frequency, nocturia	Negative	Negative	*S. montevideo*	Yes	33	16 × 12 × 12	Penicillin Terramycin	12	A
Nuttall et al. [18]	23	Dermoid cyst (infected)	India	Weight loss, anorexia, malaise, abdominal pain, vomiting, diarrhea, fever	Negative	Negative	*S. typhi*	Yes	32	20	Co-trimoxazole Metronidazole	49	A
Sharma et al. [19]	19	None	Nepal	Diarrhea, abdominal pain, fever, anorexia	Positive	Positive	*S. schwarzengrund*	Yes	37	12	Ciprofloxacin Metronidazole Ceftriaxone	14	A
This case	26	None	Japan	Diarrhea, abdominal pain, fever, nausea, vomiting	Negative	Negative	*S. stanley*	No	36	8	Ceftriaxone Azithromycin	32	A

A, alive; USA, United States of America.

Another clinical issue of *Salmonella* ovarian abscesses in reproductive-age women should be considered. Regarding superinfected endometriomas caused by *Salmonella* species, three cases (30 %) had tubal involvement (Table 2). Furthermore, regarding *Salmonella* ovarian abscesses in pregnant women, three of four cases (75 %) had tubal involvement (Table 3). Both oophoritis and tubal involvement may cause later infertility.

A limitation of this study is that positive blood and stool findings were not proven despite repeated blood and stool cultures. The low rate of *Salmonella* ovarian abscesses with endometriomas in non-pregnant and pregnant women likely correlates with the low rate of positive blood and stool cultures. However, the involvement of *Salmonella* species can be suspected according to the clinical setting (e.g., a history of improperly handled foods or close contact with animals, preceding enteritis with greenish diarrhea, and preexisting ovarian cyst), although *Salmonella* ovarian abscesses are rare. Furthermore, *Salmonella* identification can be performed from bacterial cultures of abscesses in addition to blood and stool cultures because of a higher positivity rate. Appropriate antibiotic therapy for *Salmonella* infections, considering the potential for fluoroquinolone resistance extended-spectrum beta-lactamase production, is essential to limit the effects on later fertility.

In conclusion, clinicians should pay attention to *Salmonella* species involvement in superinfected ovarian cysts, particularly in pregnant women. These patients, especially those with oophoritis or tubal involvement, should be treated appropriately to prevent later infertility, considering that *Salmonella* species may be resistant to antibiotics.

## Ethical approval

This study was approved by the institutional review board and ethics committee of Japanese Red Cross Ise Hospital (approval number: ER2020−27).

## Consent for publication

Informed consent was obtained from the patient for publication of this case report and accompanying images.

## Funding

This research did not receive any specific grant from funding agencies in the public, commercial, or not-for-profit sectors.

## CRediT authorship contribution statement

**Hirokazu Toyoshima:** Conceptualization, Methodology, Data curation, Writing - original draft, Writing - review & editing, Visualization. **Miki Hagimoto:** Conceptualization, Methodology. **Motoaki Tanigawa:** Supervision. **Hiroyuki Tanaka:** Methodology. **Yuki Nakanishi:** Methodology. **Shigetoshi Sakabe:** Supervision.

## Declaration of Competing Interest

None.
